# Differential Susceptibility to OsHV-1 in Two Geographic Populations of Ark Clam (*Scapharca broughtonii*) Based on Transcriptomic and Proteomic Analysis

**DOI:** 10.3390/microorganisms14071433

**Published:** 2026-06-30

**Authors:** Qin Liu, Shuyuan Lian, Zhuangzhuang Qiu, Yanfei Hou, Yu Zhou, Chenghua Li, Haipeng Liu, Lusheng Xin

**Affiliations:** 1 School of Public Health, Jining Medical University, Jining 272002, China; liuq@gxnzd.edu.cn (Q.L.);; 2 College of Agricultural Engineering, Guangxi Vocational University of Agriculture, Nanning 530007, China; 3 College of Marine Science, Ningbo University, Ningbo 315832, China; 4 College of Ocean and Earth Sciences, Xiamen University, Xiamen 361005, China; 5 Key Laboratory of Maricultural Organism Disease Control, Ministry of Agriculture, Yellow Sea Fisheries Research Institute, Chinese Academy of Fishery Sciences, Qingdao 266071, China

**Keywords:** *Ostreid herpesvirus* 1, *Scapharca broughtonii*, differential susceptibility, transcriptomics, proteomics

## Abstract

*Ostreid herpesvirus* 1 (OsHV-1) is a major pathogen associated with summer mortality in bivalve mollusks, but the host-side determinants of susceptibility remain poorly characterized outside oysters. In this study, we compared the responses of Chinese (CS) and Korean (KS) populations of the ark clam *Scapharca broughtonii* to experimental OsHV-1 challenge. The results showed that OsHV-1 established a markedly strong infection in the CS population but not the KS population, as indicated by high viral DNA loads, severe tissue pathology, detection of viral particles by transmission electron microscopy, broad viral transcription, and substantially high mortality. To explore the molecular basis of this contrasting outcome, we further performed comparative transcriptomic and proteomic analyses. Transcriptomic analysis identified two membrane-associated genes, *atrial natriuretic peptide receptor A* (*NPR-A*) and *tyrosine-protein kinase receptor* (*TYRO3-like*), that were more highly expressed in the susceptible CS population, suggesting that early host–virus interaction may differ between populations as a result of differentially expressed membrane molecules. Proteomic analysis identified 94 differentially abundant proteins between CS and KS populations; notably, ferritin and superoxide dismutase (SOD) were highly expressed in the less susceptible KS population, leading to stronger basal antioxidant and stress-defense capacity. Taken together, these findings definitively confirmed the significant differential susceptibility to OsHV-1 infection between the two *S. broughtonii* geographic populations and revealed that this divergent phenotype might involve both population-biased expression of candidate membrane-associated factors and differences in defense-related protein abundance. This study provides candidate markers for future disease-resistance evaluation and breeding in ark clam and offers a basis for further investigation of population-level variation in bivalve–virus interactions.

## 1. Introduction

*Ostreid herpesvirus* 1 (OsHV-1), a member of the family *Malacoherpesviridae*, is a large double-stranded DNA virus that has been widely recognized as a major cause of recurrent summer mortality in farmed bivalves, particularly under elevated water temperatures [1,2]. Since its first description in Pacific oyster (*Magallana gigas*) larvae in the early 1970s [3], OsHV-1 and its micro-variant (OsHV-1 μVar) have since been detected across Europe, Oceania, and East Asia, causing mortality rates of up to 80–100% in susceptible host populations and inflicting severe economic damage on the global shellfish industry [4,5,6]. Despite substantial progress in viral detection and genomics, the host-side determinants of susceptibility and resistance to OsHV-1 remain incompletely understood.

Important advances in this area have come from studies of selectively bred oyster families. In *M. gigas*, resistant and susceptible families have been compared under controlled challenge conditions, revealing marked differences in infection outcome [7,8]. These studies have supported the hypothesis that susceptible oysters undergo early immune suppression during the initial phase of infection, whereas resistant oysters maintain elevated expression of antiviral and immune-related genes, thereby limiting viral proliferation and secondary infection by opportunistic pathogenic bacteria [9,10,11]. However, these findings have been derived almost exclusively from studies in the Pacific oyster (*M. gigas*), the most well-characterized bivalve host of OsHV-1, and have focused primarily on post-entry host immune responses. Whether population-level differential susceptibility to OsHV-1 exists in other commercially and ecologically important bivalve species and whether variation in viral entry itself contributes to such population-level phenotypic differences remain largely unexplored.

In vertebrate virology, membrane receptors are well established as the primary gatekeepers of viral host-range and tissue tropism. Enveloped viruses exploit specific cell-surface receptors to mediate attachment and entry; the presence, absence, or polymorphism of these receptors frequently explains why closely related host populations differ in susceptibility to the same virus [12]. For example, the TAM family of receptor tyrosine kinases (TYRO3, AXL, MER) have been identified as entry receptors or entry-enhancing factors for multiple enveloped viruses, including Ebola, Dengue, and Zika viruses in mammalian systems [13,14]. In bivalves, however, the identity of membrane receptors involved in OsHV-1 entry remains poorly unknown. Given that OsHV-1 is an enveloped DNA virus, it is reasonable to hypothesize that host membrane receptors play a critical role in determining whether the virus can successfully invade host cells, implying that inter-population variation in receptor expression could contribute to differential susceptibility.

The ark clam *Scapharca broughtonii*, commonly known as the blood clam or red clam, is a commercially valuable cold–temperate bivalve widely distributed along the northwest Pacific coast, including Japan, Korea, and China [15]. Importantly, field observations and preliminary aquaculture data have indicated that the Korean population of *S. broughtonii* (KS) exhibits notably higher stress resistance, faster growth rates, and significantly lower summer mortality compared to the Chinese population (CS) [16]. These phenotypic differences suggest an underlying genetic basis for differential disease resistance. Since OsHV-1 has been implicated in summer mortality events affecting CS population ark clams, the Chinese–Korean population pair provides a natural, ecologically relevant model system for investigating the host-side molecular determinants of OsHV-1 susceptibility—one that complements the artificially selected oyster families used in previous studies.

For experimental infection studies, Koch’s postulates and their modern extensions provide a useful framework for linking pathogen exposure, pathogen detection, host pathology, and disease outcome [17]. In marine bivalve–virus systems, strict fulfillment of the classical postulates can be constrained by the difficulty of virus culture and host-specific infection models; therefore, controlled challenge experiments commonly adapt this framework by combining pathogen-free host confirmation, quantified inoculation, pathogen detection, pathology, and appropriate negative controls. Accordingly, our experimental design adapted this framework by using OsHV-1-free clams, quantified OsHV-1 inoculum, negative tissue homogenate controls, viral DNA quantification, viral transcript detection, histopathology, TEM observation, and survival analysis. In this study, we aimed to: (1) experimentally confirm the differential susceptibility of CS and KS populations to OsHV-1 through controlled infection challenge, viral quantification, histopathology, and electron microscopy; (2) employ comparative transcriptomics to identify differentially expressed genes, with particular attention to membrane receptor genes, that may underlie differential viral invasion; and (3) apply proteomic profiling to detect differentially expressed effector proteins associated with post-entry defense. By addressing both the “entry side” (receptor-mediated susceptibility) and the “defense side” (effector-protein-mediated resistance) of the host–virus interaction, this study provides an integrated framework for understanding population-level differences in OsHV-1 susceptibility and identifies candidate molecular targets for disease-resistant breeding programs.

## 2. Materials and Methods

### 2.1. Experimental Animals and Acclimation

Healthy, 2-year-old, OsHV-1-free ark clams from two geographic populations were used in this study. The CS population was from the coastal waters of Shandong Province adjacent to the Bohai Sea, China, whereas the KS population was obtained from the coastal waters of Tongyeong, Korea. The OsHV-1-free status of all clams was confirmed by qPCR before the experiments. Ark clams from these two populations were acclimated separately in 50 L tanks at a density of 20 individuals per tank for 14 d. During acclimation, the ark clams were maintained in aerated, filtered seawater (salinity, ~32‰; dissolved oxygen, 8.0 mg L^−1^; pH 8.0) at 18 °C. Seawater was renewed daily at a rate of 50%, and the ark clams were fed *Phaeodactylum tricornutum* at 1 × 10^5^ cells mL^−1^.

### 2.2. OsHV-1 Challenge and Experimental Design

For OsHV-1 inoculum preparation, mantle tissues from OsHV-1-positive ark clams stored at −80 °C were processed as previous description [18]. Briefly, tissues were homogenized on ice, centrifuged with a speed of 1000 rpm at 4 °C, and the supernatant was sequentially filtered through 5.0 µm, 2.0 µm, 0.45 µm, and 0.22 µm syringe filters. A negative tissue homogenate (NTH) was prepared in parallel from OsHV-1-negative tissues and was used as the injection control.

To assess and compare the susceptibility of CS and KS population ark clams to OsHV-1, individuals were randomly allocated to different groups following acclimation. Individuals were injected into the adductor muscle with 100 μL of OsHV-1 inoculum containing 1 × 10^6^ OsHV-1 copies as OsHV-1 infected groups. The OsHV-1 concentration in the inoculum was determined by TaqMan qPCR using the plasmid standard curve method described in Section 2.4. Control groups received 100 μL of NTH. After injection, ark clams were maintained at 18 °C. Tissue samples were collected every 24 h until 96 h post OsHV-1 inoculation. Each treatment consisted of three replicate tanks with 20 clams per tank.

### 2.3. Survival Analysis

Survival status was monitored in 50 ark clams for each group, including control and OsHV-1-infected KS/CS population groups, until 96 h post OsHV-1 inoculation. Dead ark clams were recorded every 24 h. Survival curves were generated using the Kaplan–Meier method in the software Origin 8.1.

### 2.4. OsHV-1 Quantification

Total DNA was extracted from hemocytes and mantle tissues using the DNeasy Blood and Tissue Kit (Qiagen, Hilden, Germany) according to the manufacturer’s instructions. The purity and quantity of the isolated DNA was determined using a Nanodrop 2000 spectrophotometer (Thermo scientific, Waltham, MA, USA). OsHV-1 DNA copy number was quantified by TaqMan qPCR using the primers BF and B4 together with the probe V, and the reaction setup and cycling conditions followed the protocol as described by Martenot et al. [19]. Standard curves were constructed using 10-fold serial dilutions of a plasmid containing the target sequence (10^7^–10^1^ copies μL^−1^). Viral load was expressed as the mean of OsHV-1 DNA copies per ng of total DNA for three replicates.

### 2.5. Histopathological Analysis

Mantle and gill tissues were dissected from OsHV-1-infected groups and control groups at 72 h post OsHV-1 inoculation, and then directly fixed in Davidson’s alcohol, formalin, and acetic acid (AFA) fixative for 24 h. After dehydration and embedding in paraffin wax, tissue blocks were cut into 5 µm thick sections. The sections were finally stained with hematoxylin and eosin (HE) (Beyotime, Beijing, China) and covered with DPX mounting medium (Beyotime, Beijing, China).

### 2.6. Transmission Electron Microscopy Analysis

Hemocyte and mantle tissues from OsHV-1-inoculated and control ark clams of CS and KS population were collected at 72 h post inoculation. Samples were fixed in 2.5% glutaraldehyde prepared in 0.2 M sodium cacodylate buffer at 4 °C for 24 h, post-fixed in 1% osmium tetroxide, dehydrated through a graded ethanol series, and embedded in Spurr’s resin. Ultrathin sections were further stained with uranyl acetate and lead citrate and examined using a Hitachi HT7700 transmission electron microscope (Hitachi Limited, Tokyo, Japan) operated at 80 kV.

### 2.7. RNA Sequencing Analysis

Total RNA was extracted from hemocytes of three ark clams as one repeat for each group (CS-healthy, CS-infected, KS-health and KS-infected) according to the manufacturer’s protocol of the Trizol reagent, and three repeats were set for each group. RNA quantity was evaluated using an Agilent 2100 Bioanalyzer (Agilent Technologies, Palo Alto, Santa Clara, CA, USA). mRNA sequencing libraries were constructed using the Illumina TruSeq Stranded mRNA Library Prep Kit, and paired-end sequencing was performed on the Illumina HiSeq 2000 platform.

Raw reads were filtered using fastp (version 0.18.0) to remove adaptor sequences, reads containing ambiguous bases, and low-quality reads. Clean reads were assembled de novo using Trinity and were annotated against the Nr, Swiss-Prot, GO, and KEGG databases. Differentially expressed genes (DEGs) were identified using DESeq2 with thresholds of |log_2_ fold change| ≥ 2 and adjusted to *p* < 0.05. Principal component analysis (PCA) was performed with R package (v4.4.1) models (http://www.rproject.org/, accessed on 15 May 2025) in this study. Functional enrichment analyses, including Gene Ontology (GO) terms and Kyoto Encyclopedia of Genes and Genomes (KEGG) pathways, were performed using the R package clusterProfiler (v4.6.2). The enrichment *p*-values were adjusted using the Benjamini–Hochberg false discovery rate (FDR) method, and terms with FDR ≤ 0.05 were considered significantly enriched.

### 2.8. Proteomic Analysis

Proteomic analysis was performed using hemocytes from healthy CS and KS population ark clams. Hemocytes were lysed in the lysis buffer (8M urea, 2% SDS, 1x Protease Inhibitor Cocktail) and centrifuged at 4 °C for 30 min at 13,000 × rpm. For each sample, proteins were precipitated with ice-cold acetone at −20 °C overnight. The precipitations were cleaned with acetone three times and re-dissolved in 8M urea by sonication on ice. Protein quality was evaluated by SDS-PAGE. Protein concentration was determined using the BCA assay. First, 100 μg protein for each sample was adjusted to a final volume of 100 μL with 8M urea. Then, 11 μL of 1M DTT (DL-Dithiothreitol) was added, and samples were incubated at 37 °C for 1 h. Then, 120 μL of the 55 mM iodoacetamide was added to the sample and incubated for another 20 min protected from light at room temperature to block cysteine. Proteins were tryptic digested with sequence-grade modified trypsin (Promega, Madison, WI, USA) at 37 °C overnight and then reconstituted in 0.5 M TEAB. iTRAQ labeling was performed with 100 µg of protein sample according to the manufacturer’s protocol, using an 8 × plex iTRAQ reagent (Applied Biosystems, Foster City, CA, USA).

Labeled peptide mixtures were reconstituted in buffer A (20 mM ammonium formate in water, pH 10.0 adjusted with ammonium hydroxide) and loaded onto a Waters XBridge C18 column (4.6 mm × 250 mm, 5 μm) connected to an Ultimate 3000 HPLC system (Thermo Fisher Scientific, Waltham, MA, USA). Peptides were eluted with a linear gradient of 5% to 45% buffer B (20 mM ammonium formate in 80% acetonitrile (ACN), pH 10.0 adjusted with ammonium hydroxide) over 40 min at a flow rate of 1 mL/min and 30 °C column temperature. A total of 12 fractions were collected and vacuum-dried for subsequent analysis. Each dried fraction was resuspended in 30 μL buffer C (0.1% formic acid (FA) in water) and then separated on an Easy-nLC 1000 nano-HPLC system (Thermo Fisher Scientific, Waltham, MA, USA) coupled to an Orbitrap Fusion Tribrid mass spectrometer (Thermo Fisher Scientific, Waltham, MA, USA) equipped with a nano-electrospray ion source. Then, 10 μL peptide sample was loaded onto an Acclaim PepMap C18 trap column (100 μm × 2 cm) at 10 μL/min for 3 min, followed by separation on an Acclaim PepMap C18 analytical column (75 μm × 15 cm) with a 120 min linear gradient of 3% to 32% buffer D (0.1% FA in ACN) at a constant flow rate of 300 nL/min. The mass spectrometer was operated in data-dependent acquisition mode, with full MS scans (m/z 350–1550) acquired at 120,000 resolution, followed by high-energy collisional dissociation (HCD) MS/MS scans at 30,000 resolution, with an isolation window of 1.6 Da, AGC target of 400,000, fixed first mass of 110 m/z, and 45 s dynamic exclusion.

The mass spectrometry data were transformed into MGF files with Proteome Discovery 1.2 (Thermo, Pittsburgh, PA, USA) and analyzed using Mascot search engine (Matrix Science, London, UK; version 2.3.2). Mascot database was set up for protein identification using *S. broughtonii* reference transcriptome and *S. broughtonii* database in NCBInr/SwissProt/Uniprot/IPI. Mascot was searched with a fragment ion mass tolerance of 0.050 Da and a parent ion tolerance of 10.0 PPM. The Mascot search results were averaged using medians and quantified. Proteins with fold change in a comparison more than 1.2 or less than 0.83 and unadjusted significance level *p* < 0.05 were considered differentially expressed. Proteins were annotated against GO, KEGG, and COG/KOG databases to obtain their functions. Significant GO functions and pathways were examined within differentially expressed proteins with *p*-value ≤ 0.05.

### 2.9. DEGs Validation by qPCR

Selected DEGs associated with membrane receptors and stress responses were validated by qRT-PCR. Total RNA was reverse-transcribed using the PrimeScript™II1st Strand cDNA Synthesis Kit (TAKARA, Tokyo, Japan), and qRT-PCR was performed using 2 × SYBR Green PCR Mix (Takara) on a Bio-Rad CFX Connect RealTime system (Bio-Rad Laboratories Hercules, CA, USA). *Elongation factor-1α* (*EF-1α*) was used as the reference gene. Relative gene expression was calculated using the 2^−ΔΔCt^ method [20]. Primer sequences are listed in Table 1. Each assay was performed with six replicates.

### 2.10. Statistical Analysis

All quantitative data are presented as mean ± SEM. Statistical analyses were performed using SPSS 19.0. Normality and homogeneity of variance were assessed before parametric analyses. Differences among groups were analyzed by one-way ANOVA followed by Tukey’s multiple comparison test, and *p* < 0.05 was considered statistically significant.

## 3. Results

### 3.1. The Two Geographic Populations Showed Markedly Different Outcomes After OsHV-1 Challenge

The CS and KS populations differed clearly in morphology and, more importantly, in their responses to OsHV-1 challenge (Figure 1). Quantitative PCR showed that OsHV-1 DNA increased rapidly in both hemocytes and mantle tissues of the CS population, whereas OsHV-1 DNA remained at the background level in the KS population throughout the observation period (Figure 1b). In the CS population, the copy number of OsHV-1 increased sharply at 24 h post OsHV-1 inoculation and exceeded 2.0 × 10^5^ copies per ng total DNA until 72–96 h, with mantle tissues showing the highest viral burden at the latest time point.

Survival analysis showed a similarly strong population difference (Figure 1c). The CS population began to decline after challenge and survival dropped progressively over time, reaching complete mortality by 96 h in the representative curve shown. By contrast, the Korean population maintained high survival throughout the observation period, with only a slight reduction at the final time point. Taken together, these results indicate that the CS population was markedly more susceptible to OsHV-1 than the KS population under the same challenge conditions.

### 3.2. Histopathological and Ultrastructural Analyses Revealed Severe Tissue Damage and Productive OsHV-1 Infection in the Susceptible CS Population

Histopathological analysis via hematoxylin and eosin (H&E) staining was performed on mantle and gill tissues collected at 72 h post OsHV-1 inoculation, to characterize the histopathological lesions associated with OsHV-1 infection in these two populations. In the mantle tissues of the CS population, we observed a markedly disordered and diffuse arrangement of muscle fibers, accompanied by widespread nuclear swelling in epithelial cells. As indicated by the arrows, the hematoxylin-stained (blue) nuclei appeared significantly enlarged, with paler staining and marginalized chromatin, which are typical cytopathic effects induced by herpesvirus infection (Figure 2a). In contrast, no obvious histopathological lesions in the mantle tissue of the KS population were observed. For gill tissues, the KS population exhibited no signs of infection-related damage. By contrast, the CS population presented severe gill lesions, including extensive gill filament erosion and large areas of necrosis at the frontal zone of the gill filaments, as shown in Figure 2b. These histopathological lesions were highly consistent with the high viral load and rapid mortality phenotype observed in the CS population, further confirming the markedly higher susceptibility of the CS population to OsHV-1 infection compared with the KS population.

Transmission electron microscopy provided ultrastructural evidence consistent with the population-level difference in susceptibility (Figure 3). In the KS population, no obvious virus-like particles were observed in either hemocytes or mantle tissue in the representative sections examined. In contrast, numerous viral particles were observed in both hemocytes and mantle tissue of the CS population, as indicated by the arrows in the high-magnification panels. Taken together, the histopathological lesions and ultrastructural viral particle detection consistently demonstrated that productive OsHV-1 infection was efficiently established in the CS population, causing severe tissue damage, while viral infection and the associated pathological changes were profoundly restricted in the KS population.

### 3.3. OsHV-1 Transcripts Were Predominantly Detected in the CS Population

Analysis of viral transcript abundance revealed a marked difference between the two populations (Figure 4). Viral transcripts were detected across a broad range of viral genes in the CS population, whereas transcript abundance remained consistently low across most viral genes in the KS population. Overall, the CS population showed widespread viral gene expression, while the Korean population exhibited only weak transcriptional signals. This pattern was consistent with the OsHV-1 quantification and TEM results, further supporting a substantially high viral burden in the CS population but not in the KS population.

### 3.4. Transcriptomic Analysis Revealed Pathway-Level Differences Associated with Population Background and OsHV-1 Infection

Transcriptomic analysis revealed substantial differences in pathway enrichment associated with both population background and OsHV-1 infection (Figure 5), and transcriptomic raw data were submitted to China National Center for Bioinformation (BioProject No. PRJCA062996). In the comparison between healthy CS and KS populations, KEGG enrichment analysis showed that differentially expressed genes were mainly enriched in pathways related to RNA degradation, lysosome, and metabolism related to amino acids and fatty acids. In the comparison between OsHV-1-infected and healthy CS population, differentially expressed genes were enriched in pathways related to ABC transporters, lysosome, phosphatidylinositol signaling system, metabolism of xenobiotics by cytochrome P450, mucin type O-glycan biosynthesis, N-glycan biosynthesis, carbon metabolism, and several amino acid and carbohydrate-metabolism pathways (Figure 5b), while differentially expressed genes between OsHV-1-infected and healthy KS population were enriched in pathways including neuroactive ligand–receptor interaction, ABC transporters, focal adhesion, lysosome, NOD-like receptor signaling pathway, ubiquitin-mediated proteolysis, tryptophan metabolism, arginine and proline metabolism, and several lipid- and nucleotide-metabolism pathways (Figure 5c). Overall, these results indicate that ABC transporters would be critical and conservative molecules in the response to OsHV-1 infection in both populations.

### 3.5. Differential Membrane-Associated Molecules Between CS and KS Populations

To identify candidate host factors potentially associated with susceptibility, membrane-related differentially expressed genes (Table S1) were further analyzed (Figure 6). Hierarchical clustering showed clear separation between the CS and KS populations, indicating marked differences in the expression patterns of membrane-related genes. Among these genes, Unigene0028457 (*atrial natriuretic peptide receptor A*, *NPR-A*) and Unigene0068152 (*tyrosine-protein kinase receptor*, *TYRO3-like*) were particularly notable, as both were highly expressed in the CS population, whereas they could hardly be detected in the KS population. Given their membrane-associated or receptor-related annotations, these genes were considered as candidate factors potentially associated with differential susceptibility, mediating the process of OsHV-1 invasion.

### 3.6. Proteomic Analysis Provided a Foundation for Comparative Analysis Between the Two Populations

Proteomic profiling provided an overview of the identified protein dataset and its functional composition in the healthy CS and KS populations (Figure 7), and the raw data have been deposited in the OMIX, China National Center for Bioinformation (Bioproject No. PRJCA063011). A total of 408,812 spectra were generated, from which 14,802 matched spectra, 11,004 unique spectra, 2773 peptides, 2561 unique peptides, and 810 proteins were identified. Functional classification showed that the identified proteins were distributed across a broad range of categories, with relatively high representation in post-translational modification, protein turnover and chaperones, signal transduction mechanisms, general function prediction only, translation, transcription, and intracellular trafficking, secretion, and vesicular transport. Together, these results demonstrate that the proteomic dataset provided a solid basis for subsequent comparative and functional analyses.

Differential enriched protein analysis identified 94 proteins (Table S2) that differed significantly between these two populations (Figure 8). The volcano plot revealed several proteins with marked population-biased abundance. Notably, superoxide dismutase (SOD) was among the proteins enriched in the KS population, whereas thioredoxin, oxoprolinase, CLE-related proteins, agrin, and Bm254 were more abundant in the CS population. Ferritin was also characterized and also showed a higher accumulated level in the KS population. Ferritin and SOD are functionally associated with iron homeostasis and antioxidant defense, respectively; they were considered candidate proteins potentially related to population-specific resistance.

### 3.7. Validation of DEGs by qPCR

qPCR was performed to validate the reliability of the RNA-Seq data by detecting the expression profiles of six selected DEGs. The results showed that the expression levels of stress and immune-response-related genes, including *HSP90* (*heat shock protein 90*, involved in cellular stress tolerance and immune signal regulation), *EPSTI1* (*epithelial stromal interaction 1*, participating in immune activation and antiviral response), *STAT* (*signal transducer and activator of transcription*, mediating JAK–STAT immune signaling pathway), and *IRF2* (*interferon regulatory factor 2*, regulating interferon-mediated antiviral immunity), were markedly elevated in the OsHV-1-infected KS and CS groups compared to the uninfected KS and CS groups (Figure 9). By contrast, *NPR-A* (*natriuretic peptide receptor-A*) and *TYRO3-like* (*tyrosine-protein kinase receptor*) maintained high basal expression in the uninfected CS group but decreased sharply after OsHV-1 infection in the CS group. These two membrane-associated genes might promote viral invasion, and their post-infection downregulation likely represents a host defensive response to limit viral entry; moreover, their expression was barely detectable in both uninfected and OsHV-1-infected KS groups. The qPCR expression trends of these six DEGs were highly consistent with those obtained from RNA-Seq data (Table S3).

## 4. Discussion

The present study showed that two geographic populations of *S. broughtonii* differed markedly in their susceptibility to OsHV-1 challenge. Compared with the KS population, the CS population exhibited high viral load, severe mortality, abundant OsHV-1 particles in ultrastructural observations, and broader viral transcription. By contrast, the KS population maintained low viral burden, high survival, and no evidence of viral particles or viral gene expression. Taken together, these data support the conclusion that under the same experimental conditions, the CS population was substantially susceptible to OsHV-1 while the KS population was not. This finding provides comparative evidence of OsHV-1 susceptibility in ark clams besides previously reported oysters, particularly *Crassostrea* spp. [3,21,22,23]. Our results therefore extend the context of OsHV-1 host susceptibility to *S. broughtonii* and further suggest that natural divergence among host backgrounds, including family- or population-level variation, may have important consequences for infection outcome [24,25]. More broadly, these results are consistent with the view that host background can strongly influence viral infection success in marine bivalves [22,26,27].

RNA-seq differential expression screening targeting membrane-associated transcripts identified two receptor genes with striking population-specific expression patterns: *atrial natriuretic peptide receptor A* (*NPR-A*, Unigene0028457) and *tyrosine-protein kinase receptor TYRO3-like* (Unigene0068152). Both transcripts were constitutively highly expressed in uninfected CS individuals but barely detectable in healthy KS individuals, and qRT-PCR validation fully recapitulated this RNA-seq expression trend, confirming the robustness of transcriptomic differential signals. Although these molecules could not be defined as confirmed OsHV-1 entry receptors based on the present data, their marked population-biased expression patterns strongly indicated that they might mediate critical early host–virus interactions that underpinned the divergent infection outcomes between these two *S. broughtonii* populations. *TYRO3* belongs to the evolutionarily conserved TAM receptor tyrosine kinase family, whose members are well established as entry receptors or infection-enhancing co-factors for multiple enveloped viruses in mammalian systems, via a conserved molecular mimicry mechanism that exploits the host efferocytosis pathway to mediate viral endocytosis [28]. These receptors mediate viral uptake via hijacking host endocytosis pathways, a conserved invasion mechanism applicable to enveloped OsHV-1. Our transcriptomic data showed constitutive high basal transcription of *TYRO3-like* exclusively in the susceptible CS population, which we interpreted as a population-specific molecular trait that expanded the pool of cell-surface docking sites for OsHV-1 virions before infection occurs. Upon OsHV-1 exposure, qPCR confirmed sharp downregulation of *TYRO3-like* in the CS individuals at 48 h post inoculation; this post-challenge suppression represents a compensatory host defensive response attempting to reduce available membrane receptors and restrict further viral attachment, yet the pre-existing high baseline expression in the CS individuals already enable robust initial viral invasion before transcriptional repression can take effect. In contrast, the KS individuals lacked meaningful *TYRO3-like* expression at baseline and throughout infection, removing this permissive entry factor and drastically limiting OsHV-1 adsorption and endocytosis at the earliest infection stage. KEGG pathway enrichment of membrane and endocytosis-related DEGs further supported this model: inter-population DEGs were enriched in phosphatidylinositol signaling, lysosomal trafficking, and endocytic transport pathways, all downstream signaling cascades activated by TAM receptor engagement during viral uptake.

For *NPR-A*, a membrane-bound guanylyl cyclase receptor regulating cGMP-PKG signaling, existing studies mainly focus on its core physiological and pathological roles in cardiovascular regulation, renal physiology, and cell growth control. To date, direct research on *NPR-A*-mediated virus–host interactions is extremely scarce, with no clear direct mechanism linking it to viral life cycles or host antiviral responses. While membrane-bound guanylyl cyclase family members are documented targets of some bacterial toxins, and cGMP signaling regulates immune-inflammatory responses that may indirectly impact viral infection, these associations are not specific to *NPR-A*-mediated signaling. Theoretically, as viruses often hijack host signaling pathways to support replication, it is biologically plausible that *NPR-A* may act as an accessory co-factor for OsHV-1 infection, or modulate the intracellular milieu required for efficient viral propagation via its downstream cGMP signaling [29,30]. This interpretation was further supported by our KEGG pathway enrichment analysis, which showed that differentially expressed genes between populations were significantly enriched in functional categories directly related to viral infection processes, including membrane signaling, endocytic trafficking, lysosomal function, and intracellular transport [31,32,33,34,35]. After infection, the CS individuals strongly repressed NPR-A transcription, consistent with host efforts to reverse this permissive metabolic state; however, the pre-infection high expression in the CS population already established a cellular microenvironment favorable for rapid viral proliferation, which could explain the explosive increase in viral DNA load observed from 24 h post challenge onward.

Notably, these findings currently represent high-priority candidate susceptibility factors rather than a definitively validated receptor-mediated mechanism. The assignment of functional viral entry receptors requires targeted validation via receptor blocking or gene perturbation assays, which has long been hindered in bivalve research by critical technical bottlenecks, most notably the lack of stable bivalve cell lines. Nevertheless, this study is the first to identify *NPR-A* and *TYRO3-like* as candidate mediators of OsHV-1 susceptibility in ark clams, providing key molecular targets for subsequent functional validation and supporting the development of disease-resistance evaluation and selective breeding strategies for this commercially important species.

A critical methodological clarification concerns the rationale underpinning our comparative proteomic profiling of hemocytes collected from healthy CS and KS populations, as well as how constitutive proteomic divergence in pathogen-free clams can robustly account for the stark phenotypic disparities in infection progression and host survival following viral challenge. The central mechanistic premise is that genetically fixed, population-specific disparities in basal protein abundance pre-configure intrinsic cellular defensive competence prior to pathogen encounter; such pre-established molecular profiles directly govern the host’s capacity to constrain viral propagation and mitigate virally induced oxidative stress upon infection onset. This baseline proteomic fingerprint constitutes a hardwired, heritable molecular trait that exists independent of OsHV-1 exposure and ultimately shapes the subsequent pathological lesions and divergent survival trajectories observed post challenge. Our proteomic analysis identified 94 significantly differentially abundant proteins between healthy CS and KS clams, among which ferritin and superoxide dismutase (SOD) emerged as the most functionally relevant resistance markers, with robust upregulation in uninfected KS clams, as visualized in the proteomic volcano plot (Figure 8). Both proteins are closely linked to redox homeostasis and cellular defense. Ferritin contributes to iron sequestration and cytoprotection, whereas SOD is a key antioxidant enzyme involved in reactive oxygen species detoxification and stress-responsive defense in oysters [36,37,38]. Their higher abundance in the KS population is therefore consistent with the lower-susceptibility phenotype observed in this study and with previous comparative analyses showing that oyster resistance to OsHV-1 is associated with stronger defense-related molecular responses [39]. Extensive studies in shrimp and bivalve antiviral immunity have confirmed that these two molecules synergistically mediate host defense against viral infection via two conserved core pathways. Ferritin, the primary intracellular iron storage protein, exerts antiviral effects mainly through the host “iron-withholding strategy”: it chelates free intracellular iron to create an iron-starved microenvironment that inhibits the function of iron-dependent enzymes essential for viral replication, while also enhancing host immune cell activity to restrict infection [37,40]. SOD, the rate-limiting enzyme of the cellular antioxidant system, scavenges excess reactive oxygen species induced by viral infection, maintains cellular redox homeostasis, and mitigates virus-mediated oxidative damage and apoptosis [41]. Notably, the two act synergistically: ferritin reduces free radical production at the source by limiting iron-catalyzed Fenton reactions, while SOD directly eliminates generated toxic free radicals, forming a dual immune and antioxidant barrier that underpins the OsHV-1 resistance advantage of the KS population.

Beyond their direct antiviral functions, ferritin has emerged as a promising candidate molecular marker for disease resistance breeding in bivalves, given its dual roles in innate immunity, stress tolerance, and growth regulation. Previous studies have identified a significant positive correlation between ferritin expression levels and pathogen resistance in oysters and clams, supporting its potential utility as an early screening indicator for marker-assisted selection (MAS) to accelerate the breeding of resistant varieties [42,43]. The stable differential expression of ferritin and SOD between resistant and susceptible populations in this study identifies them as core candidate markers for OsHV-1 resistance breeding in *S. broughtonii*. While challenges remain, including functional validation of gene polymorphisms and polygenic synergistic effects on disease resistance, our findings provide clear molecular targets for the selective breeding of antiviral ark clam varieties and lay a theoretical foundation for the development of immune-based prevention and control strategies against OsHV-1.

Taken together, our phenotypic, transcriptomic, and proteomic findings demonstrate that the differential OsHV-1 susceptibility between the CS and KS populations is a multi-level biological phenomenon, rather than being driven by a single molecular determinant. Specifically, the constitutive expression differences of susceptibility-associated candidate genes *NPR-A* and *TYRO3-like*, the significant upregulation of core antiviral and antioxidant effectors ferritin and SOD in the resistant KS population, and coordinated variations in pathways related to early host–virus interaction, endocytic trafficking, immune signaling and oxidative stress response, collectively shape the divergent infection outcomes between the two populations. This aligns with previous oyster-OsHV-1 studies linking disease resistance to coordinated changes across multi-omic layers rather than isolated molecular factors [10,39] and fits the established framework that host survival depends on both limiting pathogen burden and mitigating infection-associated damage [44]. A key limitation of this work is that only two geographically distinct populations were examined, and the omics-derived associations remain correlative, with functional validation required to confirm the definitive causal roles of these candidate molecules [45]. Nevertheless, the multi-omics-supported candidate molecules identified here represent promising targets for subsequent disease resistance research, population screening, and resistance-oriented breeding of ark clams, though further validation in independent populations and via functional assays such as gene perturbation is needed before they can be defined as direct determinants of OsHV-1 susceptibility or resistance [46,47]. More broadly, our analytical framework combining standardized infection phenotyping with multi-omics analysis provides an effective reference for disease resistance evaluation in non-model marine bivalves [47,48], and validated robust markers can be further applied to marker-assisted selection strategies to facilitate breeding of antiviral elite varieties for economically important bivalves [49,50].

## 5. Conclusions

This study showed that the CS and KS populations of *S. broughtonii* differed markedly in their response to OsHV-1 challenge. The CS population exhibited a more susceptible phenotype, characterized by rapid viral accumulation, severe mortality, abundant viral transcription, and viral particles in ultrastructural observation, whereas the KS population displayed a markedly weaker infection-associated phenotype. Further transcriptomic and proteomic analyses suggested that this population-level difference may be associated with membrane-related candidate genes and stronger defense-related protein signatures, especially ferritin and SOD. These findings provide a structured framework for understanding OsHV-1 susceptibility in ark clam and support the development of future resistance evaluation strategies.

## Figures and Tables

**Figure 1:**
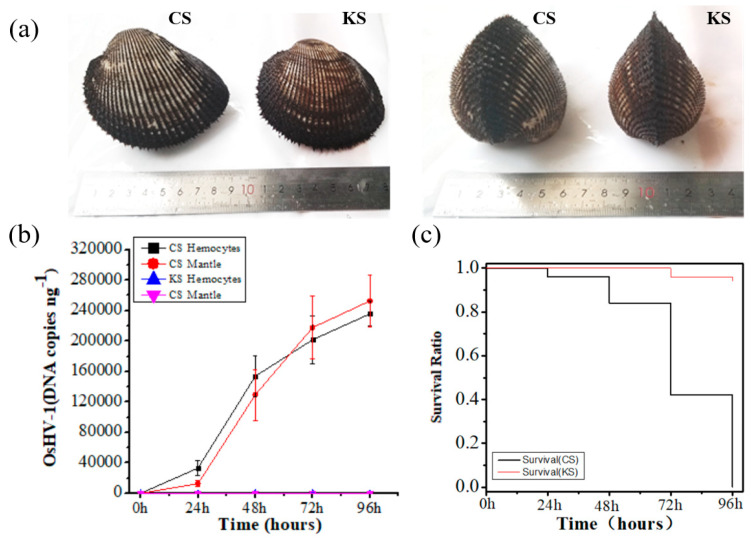
Comparison of morphology, viral load, and survival between Chinese and Korean populations after OsHV-1 challenge. (**a**) Representative images showing the external morphology of the Chinese population (CS) and Korean population (KS). (**b**) qPCR analysis of OsHV-1 DNA copy number in hemocytes and mantle tissue at different time points after challenge. OsHV-1 DNA increased markedly in both tissues of the CS population, whereas it remained at the background level in the KS population throughout the experiment. (**c**) Representative survival curves of the two populations following OsHV-1 challenge. CS, Chinese population; KS, Korean population.

**Figure 2:**
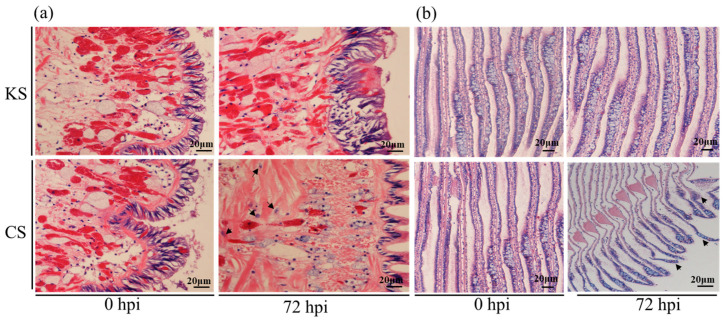
Histopathological changes in mantle and gill tissues of CS and KS populations at 72 h post OsHV-1 inoculation (H&E staining). (**a**) Mantle tissues of the KS and CS populations; arrows show swollen, pale-stained nuclei. (**b**) Gill tissues of the KS and CS populations; arrows show necrotic ulcerative lesions at the frontal zone of gill filaments. Scale bar = 20 μm.

**Figure 3:**
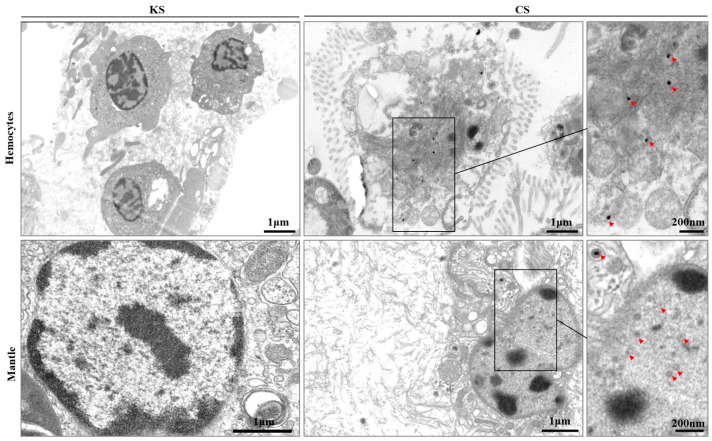
Transmission electron microscopy images of hemocytes and mantle tissues from Korean (KS) and Chinese (CS) populations post OsHV-1 challenge. No obvious viral particles were observed in KS, whereas numerous viral particles were detected in both tissues of CS population. Arrows indicate OsHV-1 particles in higher-magnification images.

**Figure 4:**
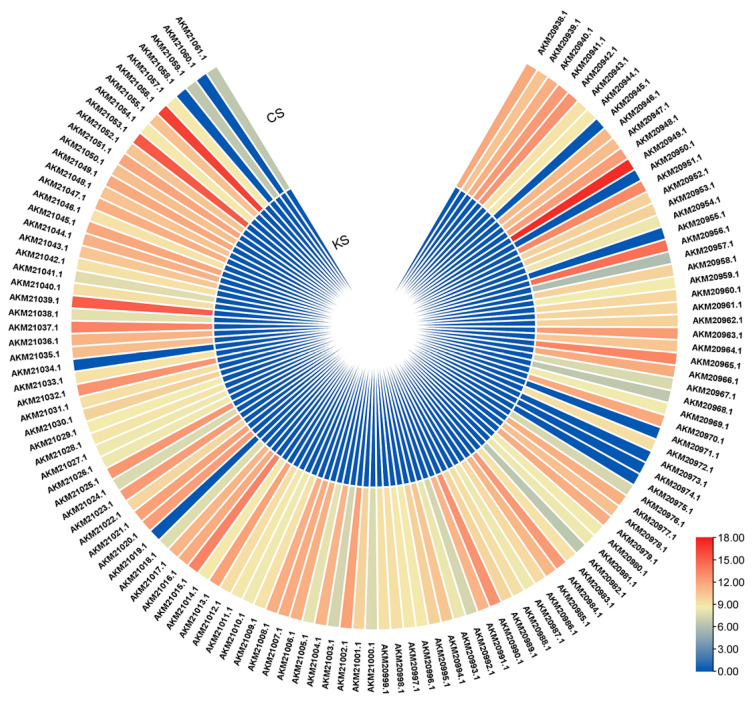
Viral transcript abundance in CS and KS populations after OsHV-1 challenge. Circular heatmap showing the expression profiles of viral transcripts in the CS and KS population. Color scale indicates relative transcript abundance.

**Figure 5:**
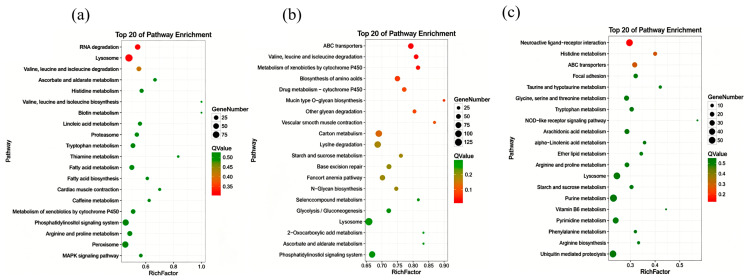
KEGG pathway enrichment analysis of differentially expressed genes associated with population background and OsHV-1 infection. (**a**) Top 20 enriched KEGG pathways for differentially expressed genes between healthy CS and healthy KS populations. (**b**) Top 20 enriched KEGG pathways for DEGs between OsHV-1-infected CS and healthy CS clams. (**c**) Top 20 enriched KEGG pathways for DEGs between OsHV-1-infected KS and healthy KS clams. Bubble size represents the number of differentially expressed genes enriched in each pathway, bubble color indicates the q value, and the *x*-axis shows the rich factor.

**Figure 6:**
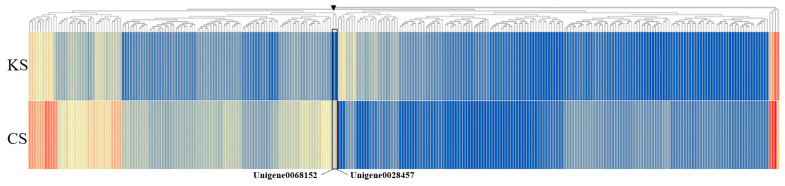
Differential expression patterns of membrane-related genes between CS and KS populations. Arrow shows the clusters (Unigene0028457 and Unigene0068152) that are only highly expressed in the CS population and could hardly be detected in the KS population (RPKM < 0.2).

**Figure 7:**
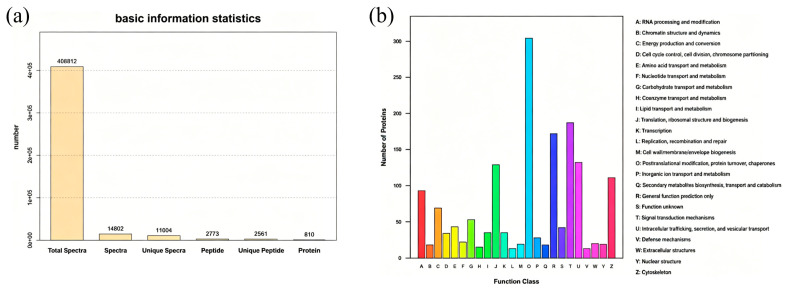
Basic statistics and functional classification of the proteomic dataset. (**a**) Numbers of total spectra, matched spectra, unique spectra, peptides, unique peptides, and identified proteins. (**b**) COG/KOG functional classification of identified proteins.

**Figure 8:**
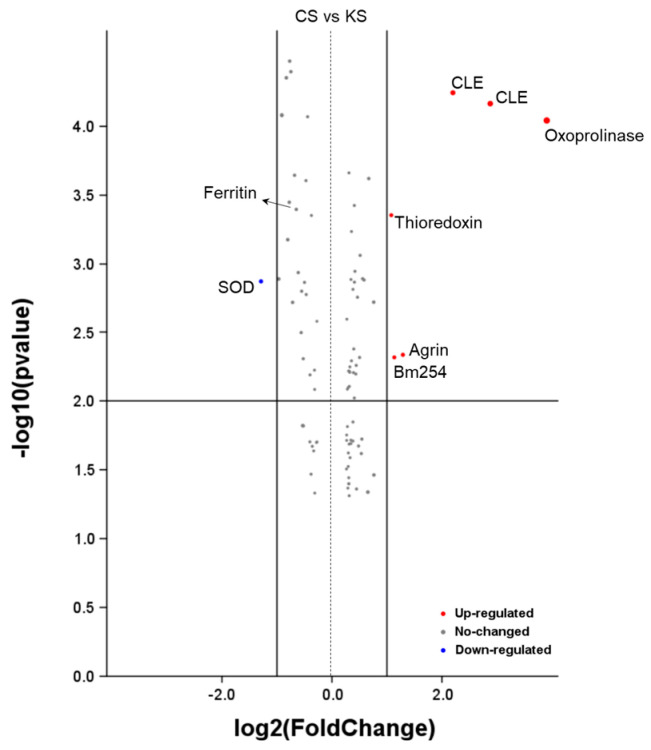
Volcano plot of differentially abundant proteins between CS and KS populations. A total of 94 proteins differed significantly between these two populations. Red dots indicate proteins enriched in the CS population with |log2 (FoldChange)| > 1, blue dots indicate proteins enriched in the KS population with |log2 (FoldChange)| > 1, and gray dots indicate proteins with |log2 (FoldChange)| ≤ 1.

**Figure 9:**
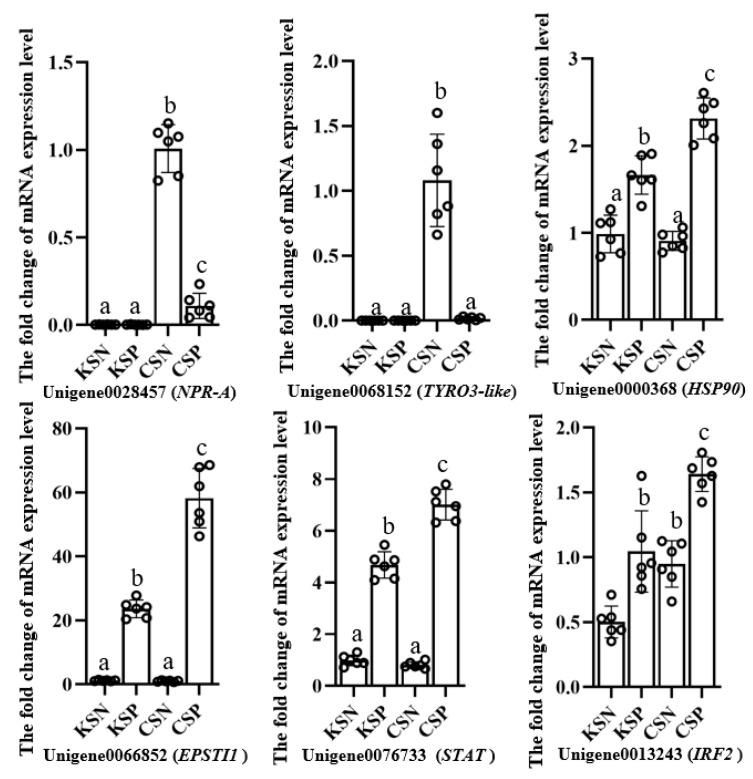
Relative expression analysis of differentially expressed genes (DEGs) by qPCR. The mRNA expression levels of 6 DEGs within the hemocytes of healthy and OsHV-1-infected KS/CS populations were determined using qPCR. KSN and CSN, healthy KS and CS population group; KSP and CSP, KS and CS population group at 48 h post OsHV-1 inoculation. All the values of the 6 DEGs’ mRNA expression levels were normalized to that of the CSN group (*n* = 6); different letters indicate significant difference with *p* < 0.05.

**Table 1:** The sequences utilized in this study.

Primer Name	Primer Sequence (5′-3′)
BF	GTCGCATCTTTGGATTTAACAA
B4	ACTGGGATCCGACTGACAAC
probe V	FAM-TGCCCCTGTCATCTTGAGGTATAGACAATC-BHQ1
*EF-1α*-F	TTAGACGCAATCCTCCCTCC
*EF-1α*-R	TAACGACGGTTCCTGGTTTG
*NPR-A*-F	CAAGCAGGACCAGAGTGAAGACC
*NPR-A*-R	TTTACCAGTTTATCGGCAGGGAG
*TYRO3-like-F*	CGTGTTCCAGCAAGTCCATCCAG
*TYRO3-like*-R	AATGTTCAAGGGCCACTAAAGCA
*STAT*-F	ACTTGGGTTAGTCTCCGAGGTAT
*STAT*-R	AGGCGTTACAGAGTCAACATACAG
*IRF2*-F	GTTGGTAGACTTGTGGTAGGAGGAT
*IRF2*-R	GAATAGATGACAATGAAAGCAGGA
*HSP90*-F	TGGTTGCTGATAGAGTCGTCGTG
*HSP90*-R	CTGATACTTCCTTGTCGCGTTCC
*EPSTI1*-F	GCTTCAGCCGAGCATCATATTGA
*EPSTI1*-R	CAGGTCAGACGGGTATGGAGGGA

## Data Availability

Transcriptomic raw data can be found at https://ngdc.cncb.ac.cn/gsub/submit/bioproject/subPRO078606/overview, accessed on 25 June 2026; proteomic raw data can be found at https://ngdc.cncb.ac.cn/gsub/submit/bioproject/subPRO077778/overview, accessed on 25 June 2026.

## Supplementary Materials

The following supporting information can be downloaded at: https://www.mdpi.com/article/10.3390/microorganisms14071433/s1, Table S1: Differently expressed membrane-associated genes; Table S2: Significantly differentially enriched proteins in the hemocytes among CS and KS populations; Table S3: The expression and annotation of all characterized genes in the RNA-seq data; Table S4. Significantly differentially transcripted genes in the hemocytes among healthy CS and KS population.
